# Serum fetuin‐A in major depressive disorder: Lower levels in patients, but no correlation with symptom severity

**DOI:** 10.1002/pcn5.70175

**Published:** 2025-08-03

**Authors:** Atsuko Ikenouchi, Rintaro Fujii, Reiji Yoshimura

**Affiliations:** ^1^ Medical Center for Dementia Hospital of the University Hospital of University of Occupational and Environmental Health Kitakyushu Japan; ^2^ Department of Psychiatry University of Occupational and Environmental Health Kitakyushu Japan

To the Editor,

Fetuin‐A (α2‐HS glycoprotein) is a plasma protein made in the liver. It helps regulate calcium and phosphate metabolism, controls inflammation, and plays a role in insulin resistance.^1^, ^2^, ^3^ These biological functions are increasingly recognized as important in the development of major depressive disorder (MDD). This study aimed to examine differences in serum fetuin‐A levels between patients with MDD and healthy controls (HC), and to explore the relationship between fetuin‐A levels and depressive symptom severity.

We recruited 30 patients with MDD, diagnosed according to DSM‐5 criteria,^4^ from the Hospital of the University of Occupational and Environmental Health, Japan. Exclusion criteria included major neurological disorders, unstable medical conditions, and cognitive impairment. All patients were undergoing antidepressant treatment (mean imipramine‐equivalent dose: 153.3 mg/day; SD = 91.6), and the average duration of illness was 6.9 years (SD = 5.9). Age‐ and sex‐matched HC participants (*n* = 30) were also enrolled. The mean age of participants was 41.5 years (SD = 9.8) in the MDD group and 44.5 years (SD = 11.7) in the HC group. The proportion of male participants was 53% in both groups. A total of 18 participants in the MDD group had comorbid physical illnesses, including hypertension, diabetes, dyslipidemia, hyperuricemia, and asthma. In the HC group, six participants presented with physical conditions such as hypertension, dyslipidemia, and asthma.

Depressive symptoms in the MDD group were assessed using the Montgomery–Åsberg Depression Rating Scale (MADRS).^5^ Serum samples were collected, processed via centrifugation, and stored at –80°C. Fetuin‐A concentrations were measured using validated biochemical assays. Group comparisons were conducted using the Wilcoxon rank sum test. To adjust for age and sex, we used multiple regression analysis. Correlations between fetuin‐A levels and MADRS scores were examined using Spearman's rank correlation.

Although unadjusted comparisons revealed no significant group difference, regression analysis adjusting for age and sex showed that fetuin‐A levels were significantly lower in the MDD group than in the HC group (mean: 249.2 μg/mL vs. 264.8 μg/mL, *p* = 0.069, adjusted *p* = 0.036, *r* = 0.235). However, no significant correlation was observed between fetuin‐A levels and MADRS scores (Spearman's rho = –0.0283, *p* = 0.8813), and regression analysis did not show a significant association between fetuin‐A and symptom severity (*p* = 0.171, 95% CI: −1.433 to 0.269).

These findings suggest that while serum fetuin‐A levels are reduced in individuals with MDD, this decrease is not associated with current depressive symptom severity. Fetuin‐A may reflect a trait‐level biological vulnerability, rather than a state‐dependent biomarker fluctuating with clinical symptoms.

Previous research supports a potential role for fetuin‐A in neuropsychiatric disorders beyond MDD. For instance, lower fetuin‐A levels have been reported in elderly patients with Alzheimer's disease, and were significantly correlated with poorer cognitive function based on Mini‐Mental State Examination scores.^6^ Fetuin‐A levels have also shown inverse correlations with tumor necrosis factor‐alpha (TNF‐α), and these associations remained significant even after adjusting for relevant covariates.^7^ These findings point to fetuin‐A's possible involvement in neuroinflammation and cognitive decline.

Given this background, fetuin‐A may also serve as a biomarker for specific subtypes of MDD—particularly those characterized by heightened inflammation or cognitive impairment, such as late‐life depression.^8^ Its role in regulating immune and metabolic function may help identify biologically distinct forms of MDD and inform tailored treatment strategies.^1^ Future research should explore the interaction between fetuin‐A and inflammatory cytokines such as TNF‐α and IL‐6 to better understand its function in depressive pathophysiology.

This study has several limitations. First, all patients were receiving antidepressant medications, which may have influenced fetuin‐A levels. Second, we did not stratify analyses by sex, despite known sex differences in immune‐inflammatory pathways associated with depression.^9^ The higher prevalence of comorbid physical illnesses among patients with depression may have influenced serum fetuin‐A levels. Clarifying the role of fetuin‐A requires larger longitudinal studies that include drug‐naïve patients, individuals without physical comorbidities, and sex‐specific subgroup analyses.

In conclusion, our findings indicate that serum fetuin‐A levels are significantly lower in patients with MDD compared to healthy controls, though no association was found with the severity of depressive symptoms. Fetuin‐A may have potential as a biomarker reflecting inflammatory or metabolic alterations in specific subtypes of MDD.

## AUTHOR CONTRIBUTIONS

Atsuko Ikenouchi contributed to the conceptualization, data curation, formal analysis, investigation, methodology, and writing of the original draft. Rintaro Fujii contributed to data curation, formal analysis, investigation, and validation. Reiji Yoshimura contributed to conceptualization, funding acquisition, supervision, validation, and writing—review and editing. All authors reviewed and approved the final version of the manuscript and agree to be accountable for all aspects of the work.

## CONFLICT OF INTEREST STATEMENT

The authors declare no conflicts of interest.

## ETHICS APPROVAL STATEMENT

This study was approved on January 19, 2022, by the Ethics Committee of the University of Occupational and Environmental Health, Japan (UOEHCRB21‐164).

## PATIENT CONSENT STATEMENT

Written informed consent was obtained from all participants.

## CLINICAL TRIAL REGISTRATION

This study is an observational study without any intervention, and does not meet the definition of a clinical trial as defined by the International Committee of Medical Journal Editors (ICMJE). Therefore, clinical trial registration is not applicable.

## Data Availability

The data that support the findings of this study are available from the corresponding author upon reasonable request.

## References

[1] Małujło‐Balcerska E , Pietras T . Adipocytokines levels as potential biomarkers for discriminating patients with a diagnosis of depressive disorder from healthy controls. J Psychiatr Res. 2024;171:163–170. 10.1016/j.jpsychires.2024.01.026 38290234

[2] Jirak P , Stechemesser L , Moré E , Franzen M , Topf A , Mirna M , et al. Clinical implications of fetuin‐A. Adv Clin Chem. 2019;89:79–130. 10.1016/bs.acc.2018.12.003 30797472

[3] Stefan N , Häring H‐U . The role of hepatokines in metabolism. Nat Rev Endocrinol. 2013;9:144–152. 10.1038/nrendo.2012.258 23337953

[4] American Psychiatric Association . Diagnostic and statistical manual of mental disorders. 5th ed. Arlington, TX: American Psychiatric Association Publishing; 2013.

[5] Montgomery SA , Åsberg M . A new depression scale designed to be sensitive to change. Br J Psychiatry. 1979;134:382–389. 10.1192/bjp.134.4.382 444788

[6] Laughlin GA , McEvoy LK , Barrett‐Connor E , Daniels LB , Ix JH . Fetuin‐A, a new vascular biomarker of cognitive decline in older adults. Clin Endocrinol. 2014;81:134–140. 10.1111/cen.12382 PMC4053483 24325554

[7] Smith ER , Nilforooshan R , Weaving G , Tabet N . Plasma fetuin‐A is associated with the severity of cognitive impairment in mild‐to‐moderate Alzheimer's disease. J Alzheimer's Dis. 2011;24:327–333. 10.3233/JAD-2011-101872 21239851

[8] Fanelli G , Benedetti F , Wang S‐M , Lee S‐J , Jun T‐Y , Masand PS , et al. Reduced plasma fetuin‐A is a promising biomarker of depression in the elderly. Eur Arch Psychiatry Clin Neurosci. 2020;270:901–910. 10.1007/s00406-019-01090-1 31863164

[9] Ramsey JM , Cooper JD , Bot M , Guest PC , Lamers F , Weickert CS , et al. Sex differences in serum markers of major depressive disorder in the Netherlands Study of depression and Anxiety (NESDA). PLoS One. 2016; 11: e0156624. 10.1371/journal.pone.0156624 27232630 PMC4883748

